# Virtual Surgical Planning and Piezoelectric Surgery in Tumor Extirpative Surgery Aimed at Inferior Alveolar Nerve Preservation

**DOI:** 10.1155/2017/4397178

**Published:** 2017-12-31

**Authors:** Eric L. Chung, Faizan Alawi, Anh D. Le, Rabie M. Shanti

**Affiliations:** ^1^Department of Oral and Maxillofacial Surgery and Pharmacology, University of Pennsylvania School of Dental Medicine, Philadelphia, PA, USA; ^2^Department of Pathology, University of Pennsylvania School of Dental Medicine, Philadelphia, PA, USA; ^3^Department of Otorhinolaryngology, Head and Neck Surgery, University of Pennsylvania School of Medicine, Philadelphia, PA, USA

## Abstract

A myriad of extirpative surgical protocols for the management of benign tumors of the jaws have been presented in the literature. Through significant advancements in computer-aided design and computer-aided manufacturing (CAD/CAM) technology and surgical instrumentation, today surgeons have at their disposal robust technology-driven techniques that are aimed at improving surgical outcomes. Our goal is to investigate the benefits of implementing virtual surgical planning (VSP) in conjunction with piezoelectric surgery (PES) to ensure success while minimizing the risk of complications during extirpation of tumors of the mandible. This case report describes the successful extirpation of an ossifying fibroma of the mandible in an adult patient using both VSP and PES.

## 1. Introduction

Ossifying fibroma (OF) is a rare and benign fibro-osseous neoplasm found most commonly among the bones of the craniomaxillofacial skeleton. Traditionally, surgical treatments of the OF vary from enucleation and curettage versus resection with high-speed surgical handpieces (rotating burs and reciprocating saws), putting neighboring soft tissue and vital structures at risk for iatrogenic injury. Such procedures were limited in their accuracy in that they required assessment of two-dimensional imaging prior to surgery and performing extirpative procedures with little intraoperative guidance. However, today surgeons are able to employ highly sophisticated technologies that can optimize design of osteotomies for extirpation of bony tumors while still considering adjacent critical structures. Furthermore, the surgical armamentarium of surgeons has expanded with instrumentation that allows for further protection of these critical structures.

## 2. Case Presentation

**Figure 1:**
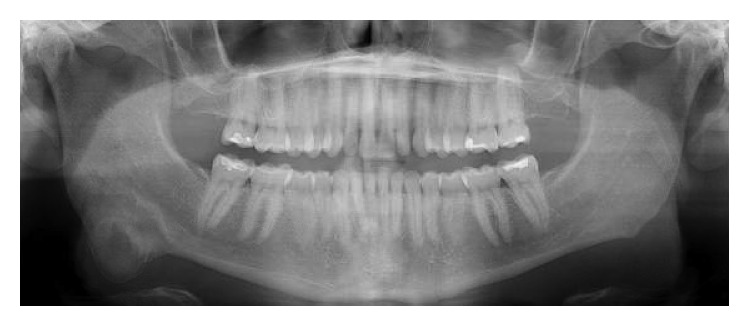
Preoperative orthopantomogram revealing a mixed (radiolucent-radiopaque) lesion, the inferior border of the mandible in the right angle region.

**Figure 2:**
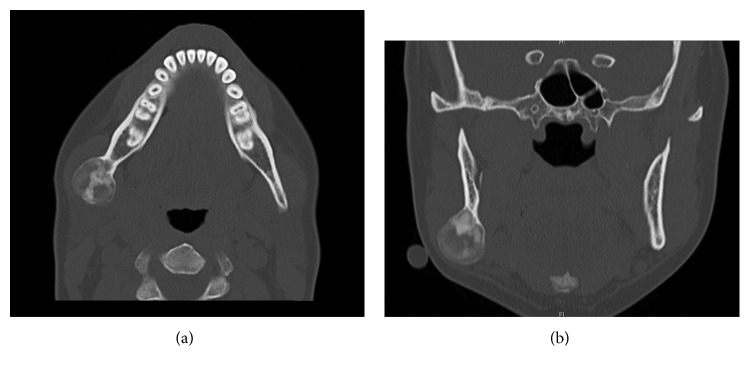
(a) CT axial cross section and (b) CT coronal cross section of an expansile mixed lesion of the right angle region of the mandible.

A 39-year-old woman initially presented to the office of the senior author (Rabie M. Shanti) for evaluation of an asymptomatic right-sided facial swelling of 2-month duration. The patient's past medical history was noncontributory. On head and neck examination, the patient's face was notable for slight asymmetry, with mild swelling of the lower right side of the face along the angle of the mandible that was consistent with a firm and nontender palpable mass with well-defined margins. Normal cranial nerve exam was noted. There was no evidence of palpable cervical or submandibular lymphadenopathy. Orthopantomogram showed a large mixed well-circumscribed bony lesion along the angle of the right mandible that extended approximately 1 cm beyond the inferior border of the mandible (Figure 1). No involvement of dentition or violation/displacement of the mandibular canal was noted. Computed tomography (CT) scan of the maxillofacial region identified a 22 mm × 24 mm mixed (radiolucent-radiopaque), well-circumscribed, expansive lesion at the right mandibular angle region, not involving the dentition (Figures 2 and 2). Subsequently, an incisional biopsy via transoral route was performed. The specimen was microscopically diagnosed as a benign fibro-osseous lesion that in correlation with radiographic imaging favored a diagnosis of ossifying fibroma.

**Figure 3:**
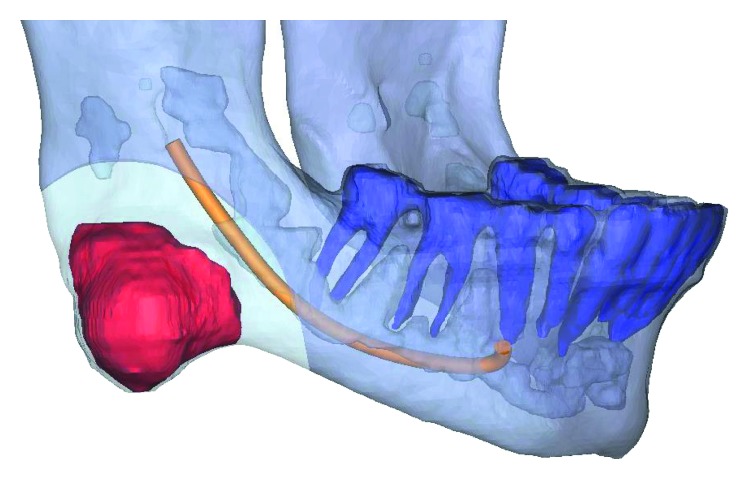
Virtual surgical plan allowing for 5 mm surgical bony margin surrounding the tumor, indicating overlap between surgical margin and mandibular canal.

Due to the tumor involving the inferior border of the mandible, a recommendation of tumor resection was made. Virtual surgical planning was performed to allow for the fabrication of a cutting guide to ensure a 5 mm surgical bony margin. However, such a margin was noted to overlap with the mandibular canal placing the patient at high risk for transection of the inferior alveolar nerve during tumor extirpation (Figure 3). Therefore, due to the high risk for inferior alveolar nerve injury, a combination of piezoelectric surgery and virtual surgical planning was utilized to allow for preservation and transposition of the nerve, while also maintaining a 5 mm surgical bony margin.

**Figure 4:**
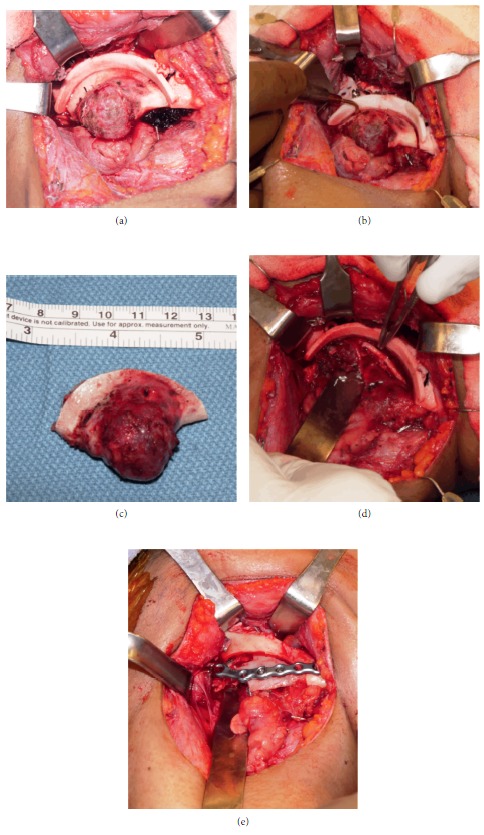
(a) Intraoperative photograph following exposure of tumor and application of custom tumor cutting guide. (b) Piezoelectric handpiece used to perform osteotomy. (c) Resection specimen. (d) View of the inferior alveolar nerve following completion of resection. (e) Autogenous anterior iliac crest bone graft in place.

The patient subsequently underwent right marginal mandibulectomy with 5 mm surgical margin via transcervical route utilizing a customized cutting guide for guidance of osteotomy (Figure 4). Piezoelectric surgical system was used to perform the osteotomy (Figure 4), and following its completion the inferior alveolar nerve was noted to be intact (Figure 4). However, a small superficial laceration to the nerve was noted and was immediately addressed with a 9-0 nylon epineurial suture. Following tumor extirpation (Figure 4), a 2.0 mm reconstruction plate was secured to the mandible, and an autogenous anterior iliac crest bone graft was used to reconstruct the mandibular defect (Figure 4).

**Figure 5:**
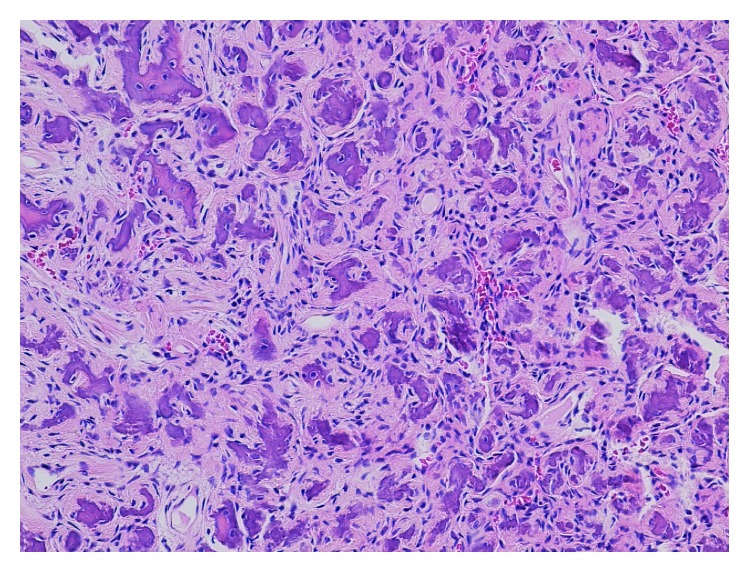
Histologic imaging of gross specimen. Histopathology of the tumor comprised numerous small, round ossicles embedded in cellular fibrous stroma, and occasional osteoclast-like giant cells are identified. The lesion is well demarcated from the surrounding bone. No increased mitotic figures or necrosis is seen. Benign fibro-osseous lesion, consistent with ossifying fibroma of the jaw (hematoxylin and eosin, 100x).

Final histologic analysis indicated a diagnosis of ossifying fibroma (fibrous connective tissue with islands of calcifications similar to bone and cementum) (Figure 5). The patient currently is 8 months' status postsurgery and has normal neurosensory function of the right inferior alveolar nerve distribution with a pinpoint area of hypoesthesia along the right lateral lip vermillion border measuring 2 × 2 mm. Tooth #31 remains vital and asymptomatic. To date, there have been no clinical and/or radiographic signs of local recurrence.

## 3. Discussion

“Benign fibro-osseous lesions” (BFOLs) is a generic term used to histologically classify bony lesions replaced with benign connective tissue [1]. BFOLs encompass a myriad of lesions and account for 17.6–39.9% of all lesions within the oral cavity [2, 3]. Among BFOLs, ossifying fibroma (OF) is a rare and benign fibro-osseous neoplasm found most commonly among the craniofacial bones. Over 70% of OF lesions of craniofacial skeleton involve the maxilla and mandible, with the latter being the most common [4]. Nonetheless, rare cases of OF have been reported in the long bones, nasal cavity, paranasal sinuses, temporal bone, frontal bone, and skull base [4–6]. It is most commonly observed in females during the second to fourth decade of life as an asymptomatic and asymmetrical mandibular buccal and/or lingual expansion and swelling [7, 8]. Despite their normal slow growth process, significant growth potential can be observed if these lesions are left untreated [9]. Although rare, a more aggressive variant, juvenile ossifying fibroma (JOF), can be seen among younger patients (usually younger than 15 years of age). The pathogenesis of OF is largely unknown and has been tied to trauma, infection, neoplastic, and developmental origins. The origin of OF was originally thought to arise from undifferentiated cells of the periodontal ligament tissues [10]; however, reports of histologically similar lesions presenting in other craniofacial bones aside from the maxilla and mandible suggest otherwise.

Traditionally, surgical treatments of OF include enucleation and curettage versus resection with delayed or immediate reconstruction to restore form and function [11]. Small, well-defined lesions can be treated with conservative curettage or enucleation with a favorable prognosis. Due to the morbidity associated with resection of portions of the jaw bones, such extirpative procedures should only be reserved for large, aggressive, lesions involving the inferior border of the mandible and/or extending into the maxillary antrum and/or nasal cavities [11]. Conventionally, extirpative surgical techniques involve the use of traditional bony surgery armamentarium (high-speed surgical handpieces with rotating burs, reciprocating saws, mallets, and chisels), which are a low cost and readily available option in the rapid nonselective cutting of hard tissue at the expense of generating excessive heat and bone fragments, putting surrounding soft tissue vital structures at risk for iatrogenic injury. Due to the inherently compact anatomy of the head and neck, vital anatomic structures are at risk of partial or complete injury during tumor extirpation. Complications that are associated with extirpative procedures of the jaws include but are not limited to incomplete resection, damage to nearby vital structures, severe hemorrhage, hematoma, infection, seroma, malocclusion, dehiscence, and iatrogenic fracture [12].

With the advancement of medical technology, new surgical instruments have been developed in attempts to minimize iatrogenic injury, indirectly increasing the weight of surgeon experience and the presence of nontypical anatomy. Methods to reduce the incidence of iatrogenic injury to adjacent structures include VSP and PES. VSP allows visualization of surrounding and deep anatomy, construction of a comprehensive preoperative plan, and more precise osteotomies and restoration via use of intraoperative surgical cutting jigs as well as custom prebent reconstruction plates. PES was first described in the 1880s with claims of preserving nerves and vessels by controlled and selective cutting of hard tissue [13, 14]. PES utilizes electrical currents to create oscillations of ultrasonic frequency to exclusively cut mineralized tissue while preserving neighboring soft tissue structures. Together, these techniques minimize complications while allowing for more precise osteotomies. Unfortunately, today rotary instruments, such as surgical handpieces with a rotating bur or reciprocating saw, are still the mainstay in head and neck extirpative surgery. Therefore, it is our hope to advance head and neck surgical techniques by presenting a successful extirpative surgery case using these robust technologies.

OF lesions should be treated as conservatively as possibly to preserve aesthetics, occlusion, and function. Small and well-defined OF lesions are typically excised by curettage and enucleation; however, large expansile lesions with aggressive patterns require extirpative surgery with healthy margins (>3 mm) and aesthetic recontouring [11, 15, 16]. The recurrence rate of OF ranges from 0% to 28% with most recurrences occurring among patients treated with curettage alone. Recently, Titinchi and Morkel proposed a protocol for the surgical management of OF lesions (enucleation versus curettage versus resection with reconstruction) [11]. Based on this protocol, our patient underwent resection with reconstruction due to inferior border of mandible involvement. Titinchi and Morkel recommended resection with > 3 mm to <5 mm margins would have caused violation of the IAN canal, so preoperative VSP and utilization of PES played a key role in achieving a successful extirpative surgery [11].

A successful extirpative surgery is classified as complete removal of the infected organ or tissue with negative margins; however, preservation of major anatomical structures should always be attempted. There are many complications that may arise from extirpative surgery. Although rare, these complications should be avoided at all costs. Traditionally, rotating burs and reciprocating saws are used to nonselectively cut bone during resection surgeries, putting surrounding soft tissue structures at significant risk of injury. With the constant advancement of medicine, surgical techniques, and technology, all options should be considered during the preoperative planning stage to help reduce any predictable risks of complications during surgery.

The piezoelectric effect states that when a mechanical stress is applied to certain ceramics and crystals, an electrical charge is generated. First described by Jacque and Pierre Curie in the 1880s, piezosurgery evolved from exploiting the piezoelectric effect and utilizing ultrasonic microvibrations to cut through mineralized tissue [17]. These ultrasonic vibrations create a cavitation phenomenon (mechanical cutting that occurs exclusively on mineralized tissue) when made in contact with bone [14]. PES also allows for increased osteotomy precision due to the predictable force and rate of cutting, while conventional drills and saws are greatly affected by the density of the mineralized tissue. Cavitation method and vibrations produced by piezo handpieces cause blood to naturally be removed, allowing for a cleaner surgical field. This contrasts with conventional rotating burs that create bone dust to accumulate and blood from moving in and out of the surgical area. Altiparmak et al. documented a 0.014% chance of damaging nearby dentition and a statistically significant reduction of postoperative skin and mucosa paresthesia when utilizing PES compared with conventional techniques for surgical graft harvesting of the mandible [18]. Use of the piezoelectric technique has also been reported to cause less postoperative pain, swelling, and recovery time [17]. Several studies have shown that bone harvested with a piezoelectric handpiece had a better chance of success when grafted than bone harvested with a round bur on a traditional surgical handpiece. This was likely due to the ability to preserve osteocytes, causing an overall increased number of remaining osteocytes with less occurrence of nonvital bone. In the present study, no lesions of the mandible nerve were detected with piezosurgery, whereas surgery with rotary instruments resulted in 8% hypesthesia [17]. Schaeren et al. reported that direct exposure of a nerve to piezosurgery would preserve the perineural sheath and never transect or dissect the nerve; therefore, any nerve contacting the piezoelectric handpiece tip should theoretically be able to regenerate [19]. Although the advantages of PES are widely accepted by oral and maxillofacial surgeons, it is seldom used during head and neck surgery. Despite the numerous advantages of piezoelectric surgery, it has been largely criticized for longer intraoperative time. However, a study by Landes et al. discussed the application of PE surgery in 90 orthognathic surgery resulted in similar surgery time and reduced blood loss when comparing to conventional techniques [20]. This study explained that with increased operator PES experience and utilization of the Epker method (creating grooves in bone with a layer of cortical bone using a fissure bur, then pressing the piezoelectric tip into these grooves to cut the cortical bone completely), this disadvantage can be largely prevented [21]. As with all surgical techniques, the precision and overall outcome may vary depending on surgeon's experience and case selection. VSP is an effective tool that allows residents/fellows, attendings, and biomedical engineers a platform to discuss preoperative anatomy, surgical techniques, and potential challenges to help minimize complications while simultaneously maximizing outcomes. VSP helps to control unpredictable variables, optimizes efficiency, and provides an ideal reconstruction plan that can be transferred intraoperatively via surgical cutting guides [22]. Fabrication of intraoperative surgical cutting guides allows for improved accuracy and decreased intraoperative time [23]. Zhang et al. described an overall mean linear difference of 0.81 mm (0.71 mm for the maxilla and 0.91 mm of the mandible) among 30 consecutive double-jaw orthognathic surgery patients planned with VSP (occlusal splints and cutting jigs) [24]. Roser et al. reported accuracies of 2.00 ± 1.12 mm among 19 mandibular osteotomies and 1.30 ± 0.59 mm among 44 independent fibula osteotomies [23]. Roser et al. stated that the accuracy of fibula osteotomies was largely based by the placement of the surgical guide which is ultimately dictated by the location of the flap's perforator vessels [23]. Several studies utilizing VSP for maxillary and mandibular resection surgery with a fibula free flap reconstruction indicated an increased accuracy among fibula osteotomies; likely due to the relative accessibility of long bones in comparison to facial bones. Bernstein et al. reported that the median distance among 224 unnavigated and 244 navigated osteotomies was 2.1 mm and 1.2 mm, respectively [25]. The option of VSP may increase a surgeon's confidence and overall likelihood of treating larger and more complex cases. However, it is important to note that surgeons who are unfamiliar with VSP technology and surgeons who possess less surgical experience may ultimately produce larger variations when comparing pre- and postoperative results [22].

## 4. Conclusion

In general, many studies have shown the superiority of PE surgery over conventional surgery and the excellent predictability when using preoperative VSP with intraoperative surgical cutting guides. The overall use of VSP and PE surgery is undoubtedly superior to traditional unguided surgery and the use of conventional rotary handpieces and reciprocating saws. With continued use and study, our long-term goal is to develop new standards for the management of head and neck extirpative surgeries. In conclusion, VSP and PE surgery should always be considered whenever performing surgery in a compact anatomical zone such as the head and neck region.
